# Appendicitis as a cause of intestinal strangulation: a case report and review

**DOI:** 10.1186/1749-7922-4-34

**Published:** 2009-10-10

**Authors:** Laxminarayan Bhandari, PG Mohandas

**Affiliations:** 1 Dept. of Surgery, Calicut Medical College, Kerala, India

## Abstract

Intestinal obstruction is a common surgical emergency caused by varied conditions. Appendicitis as a cause is both uncommon and unsuspected. Strangulation of intestine caused by appendicitis is extremely rare with very few cases reported in literature. The diagnosis of such a condition is possible only on table, with CT having questionable value. This is a very rare and dangerous complication of a very common disease which can easily be overlooked. Every emergency surgeon needs to be aware of such a possibility.

We report a case of a 24 year old male presenting with classical features of intestinal obstruction. On laparotomy strangulated bowel was seen and appendix was found to be the cause. Although we obtained a history of appendicitis in this patient, it was not correlated to the present condition due to the rarity of such a scenario. We reviewed literature to find similar cases reported in the past.

## Background

Intestinal obstruction is a common surgical emergency caused by varied conditions. Appendix as a cause of intestinal obstruction is uncommon and not usually suspected. Although it was described as early as 1901, very few reports are available which do a comprehensive review [1]. Intestinal strangulation caused by appendix is extremely rare with very few cases reported. Pre-operatively it is very difficult to diagnose this condition. The diagnosis is always made at the time of laparotomy. The treatment varies from appendicectomy to intestinal resection or even right hemicolectomy.

We are reporting a case of intestinal strangulation caused by appendicitis, for which appendicectomy was done. This is a very rare complication of an extremely common disease. We reviewed the literature to find out about appendix producing intestinal obstruction in general and intestinal strangulation in particular.

We have included a comprehensive discussion about appendicitis producing intestinal obstruction with regards to its various pathological types, different clinical presentations, diagnosis and management.

## Case report

A 24 year old man presented with on and off fever and diffuse abdominal pain since one week. He also had constipation, vomiting and abdominal distention since two days.

He was apparently normal a week ago when he developed abdominal pain for which he visited a peripheral hospital. An Ultrasonography (USG) abdomen was done, which revealed the possibility of a mild appendicular inflammation. He was treated with oral antibiotics and analgesics following which his abdominal pain subsided. Few days later he developed abdominal distention and vomiting.

On examination he was afebrile and vitals were stable. Abdomen was soft, distended and non tender. Free fluid was present in peritoneal cavity and bowel sounds were absent. Routine blood investigations were normal except for leucocytosis of 30,400 with neutrophilia. Plain X-ray of abdomen showed dilated jejunal and ileal loops with multiple air-fluid levels. Paracentesis yielded hemorrhagic ascetic fluid. USG abdomen revealed gross ascites and thickened bowel wall with absent peristalsis. Contrast enhanced CT abdomen showed small bowel obstruction and massive ascites.

Meanwhile patient was kept nil per oral with nasogastric aspiration. He was started on prophylactic intravenous antibiotics and analgesics. On reassessment patient's condition remained unaltered. A diagnosis of mechanical intestinal obstruction of unknown etiology was made and he was scheduled for emergency laparotomy.

**Figure 1:**
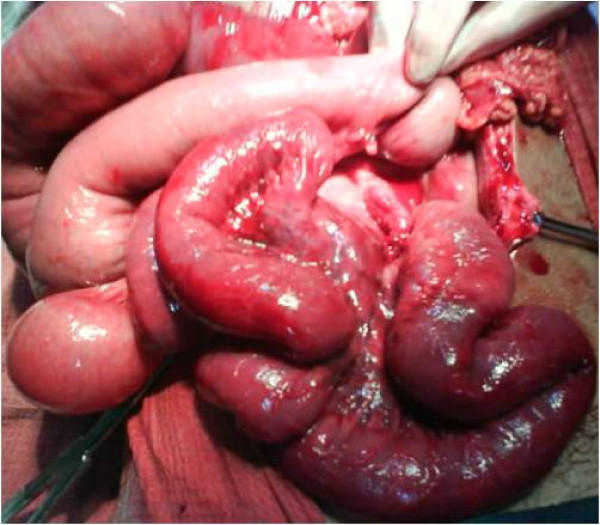
**On table picture showing dilated proximal intestinal loops and a part of Ischemic ileum**. Note the clear line of demarcation between healthy and involved ileum.

**Figure 2:**
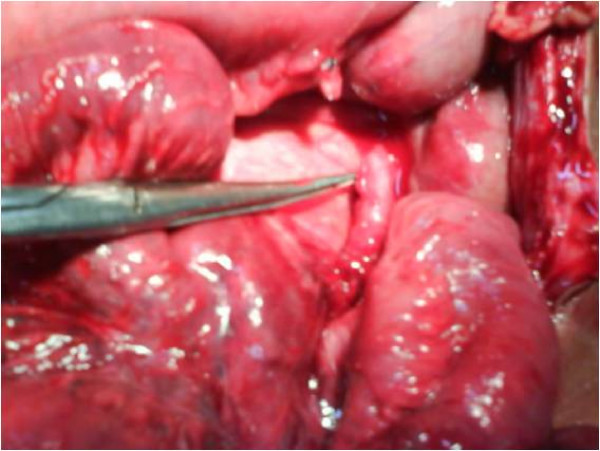
**Higher magnification picture showing the band which had produced strangulation**. This band composed predominantly of appendix and in part by omentum (not shown in this picture). Note the area of attachment of the band on distal ileum.

**Figure 3:**
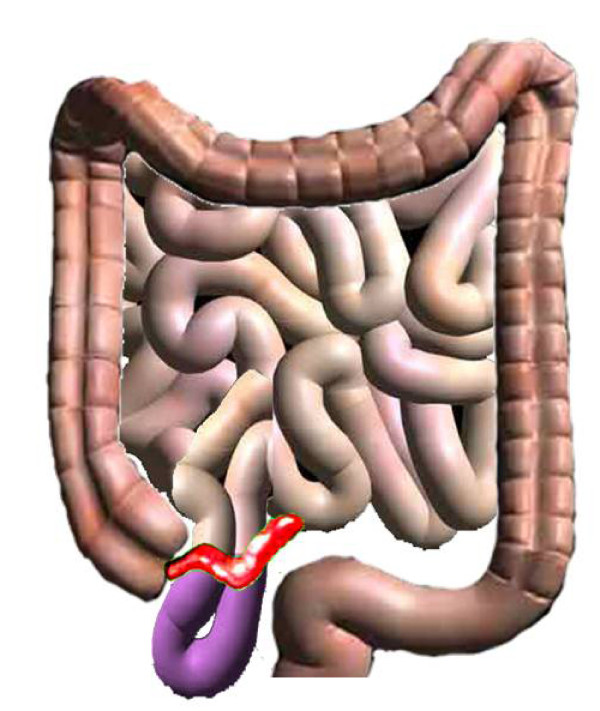
**Diagrammatic representation of the process of intestinal strangulation**. Appendix adhered to distal ileum producing a window underneath. Part of bowel herniated through the gap and underwent strangulation.

Abdomen was opened with a midline vertical incision. Three litres of hemorrhagic fluid was drained. Dilated jejunal loops were seen. These loops were traced up to a segment of ischemic ileum. The ileal segment was strangulated by a band composed of inflamed appendix and omentum (Fig 1 &2). The band was running from caecum to ileum producing a window underneath. Through this window the intestine had protruded (Fig 3).

The attachment of the band was released from the ileum and omentum, following which appendicectomy was done. The bowel was found viable and hence no resection was needed. Post op period was uneventful and patient was discharged on 7^th ^day. Histopathology report confirmed acute appendicitis. On three month follow up he is doing well.

## Discussion

Appendicitis causing intestinal obstruction was described as early as 1901, when Lucius Hotchkiss read at the meeting of New York surgical society, three successful surgeries for intestinal obstruction due to appendicitis [1]. In 1908, Forbes Hawks divided them into mechanical, septic and a combination of the two [2].

After a thorough review of literature, we found that the underlying pathology in intestinal obstruction caused by appendicitis could be classified into:

1. Adynamic

2. Mechanical (without strangulation)

3. Strangulation of intestine

4. Intestinal obstruction due to mesenteric ischemia.

Adynamic type of intestinal obstruction is due to the local paralytic ileus occurring as a result of appendicular inflammation spreading to the adjacent bowel wall. This is the most common type, seen in 1-5% of appendicitis.

Mechanical intestinal obstruction without strangulation occurs as a result of kinking, compression or traction of the small bowel trapped in an appendicular mass or abscess. These can be managed conservatively as the obstruction should resolve with the resolution of the mass. However in some cases, minimal obstruction may persist which can turn into acute intestinal obstruction when a secondary pathology occurs months to years later [3].

The first case of small bowel strangulation caused by appendix was described by Naumon in 1963 [4]. Strangulation can be due to the appendix wrapping around the base of a bowel loop, or when inflamed appendix adheres to caecum, small intestine or posterior peritoneum and a part of the bowel herniates through the gap. This is a rare occurrence with only ten other cases reported in literature. [4-11]

Intestinal obstruction occurring as a result of mesenteric ischemia produced by appendix is the rarest type with a sole case described by Gupta S. in 1969 [7]. The inflamed appendix was adhered to the mesentry near the iliocolic artery causing thrombosis and gangrene of terminal ileum.

As to why appendix would adhere to adjacent structures, we have to know that the appendix is a mobile organ with many variations in its normal position. During the initial event of appendicular inflammation, it would get adhered to surrounding structures producing various pathologies mentioned above. Increased length of appendix logically seems to predispose to such an event. [10]

Although the pathology may vary, clinically it is not possible to determine the exact type of intestinal obstruction present. Clinically these patients can be classified into two types:

1) Predominant features of appendicitis with some evidence of intestinal obstruction: In this group of patients, intestinal obstruction occurs during the phase of active appendicitis. Hence the cause is likely to be mechanical or adynamic. However, as mentioned by Assenza, strangulation too may be seen in the acute phase [10].

2) Patients with Acute intestinal obstruction, on evaluation/laparotomy found to have appendicitis as the cause. In this group, there may or may not be a history of appendicitis. Intestinal obstruction dominates the clinical picture and may completely obscure the underlying appendicular disease. Appendicitis should therefore be considered in cases of mechanical intestinal obstruction of unknown cause, especially in the elderly.

Role of CT in detecting appendix as the cause of intestinal obstruction is questionable. During the phase of active appendicular inflammation there may be appropriate CT findings. However these findings may not be present in patients who develop intestinal obstruction after the resolution of appendicitis. Thus pointing out appendix as the cause would not be possible. However CT is very useful to detect bowel ischemia, intestinal obstruction and ascites when present.

Early diagnosis and intervention is very important in strangulation of bowel. Whenever features of intestinal obstruction predominate, we recommend using a mid line vertical incision as the exact pathological type cannot be determined. Mc Burney's incision may suffice if the obstruction is Adynamic or Mechanical. However it would be inadequate and even disastrous if strangulation or mesenteric ischemia is present, as these are likely to be overlooked [3].

In case of intestinal obstruction without known cause, as with the second group, midline vertical incision is definitely the approach of choice.

There is no material available as to the role of laparoscope either with the diagnosis or management of intestinal obstruction due to appendicitis. It may be useful since it is diagnostic as well as therapeutic. There is less tissue handling; better cosmesis and a shorter post op stay [12].

## Conclusion

Intestinal obstruction due to appendicitis may be of 4 types: Adynamic, Mechanical, Strangulation and due to Mesenteric Ischemia. Clinically and radiologically it may not be possible to differentiate these types.

Clinically the presentation may be predominantly appendicitis or predominantly intestinal obstruction. In the second group it is important to rule out appendicitis by careful re-evaluation. Role of CT in detecting appendix as the cause of intestinal obstruction is questionable.

Midline vertical incision would be the approach of choice whenever features of intestinal obstruction predominate, even if appendicitis is known to be the etiological agent.

Whenever there is intestinal obstruction associated with acute appendicitis, it may not always be Adynamic and the rarer and more dangerous forms should always be kept in mind.

## Consent

Written informed consent was obtained from the patient for publication of this case report and any accompanying images. A copy of the written consent is available for review by the Editor-in-Chief of this journal.

## Abbreviations

CT: computerized tomography; USG: ultra sonogram.

## Competing interests

The authors declare that they have no competing interests.

## Authors' contributions

BPL participated in the admission and the care of this patient, the conception, the design, data collection and interpretation, manuscript preparation and literature search.

MPG participated in the admission and the care of this patient, the conception, the design, data collection and interpretation, manuscript preparation and literature search. All authors read and approved the final manuscript.
